# Dibutyl 2,2′-bipyridine-4,4′-dicarboxyl­ate

**DOI:** 10.1107/S1600536809003997

**Published:** 2009-02-06

**Authors:** Qianli Li, Rufen Zhang, Yang Shi

**Affiliations:** a College of Chemistry and Chemical Engineering, Liaocheng University, Shandong 252059, People’s Republic of China; b Department of Chemistry, Liaocheng University, Liaocheng 252059, People’s Republic of China

## Abstract

In the title compound, C_20_H_24_N_2_O_4_, the mol­ecule lies on a centre of symmetry and is approximately planar (r.m.s. deviation= 0.013 Å for 26 non-H atoms). The carboxyl­ate group is inclined slightly to the neighbouring pyridine ring, forming a dihedral angle of 4.37 (2)°. The mol­ecules form stacks with an inter­planar separation of 3.547 (1) Å.

## Related literature

For related structures, see: Stocco *et al.* (1996 ▶); Tynan *et al.* (2003 ▶); Fujihara *et al.* (2004 ▶).
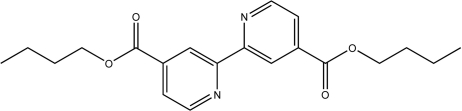


## Experimental

### 

#### Crystal data

C_20_H_24_N_2_O_4_
*M* *_r_* = 356.41
Monoclinic, 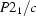

*a* = 7.4183 (9) Å
*b* = 8.2829 (10) Å
*c* = 15.375 (2) Å
β = 93.273 (1)°
*V* = 943.2 (2) Å^3^
*Z* = 2
Mo *K*α radiation
μ = 0.09 mm^−1^
*T* = 298 (2) K
0.40 × 0.30 × 0.15 mm

#### Data collection

Bruker SMART CCD diffractometer
Absorption correction: multi-scan (*SADABS*; Sheldrick, 2003 ▶) *T*_min_ = 0.966, *T*_max_ = 0.987
4552 measured reflections
1654 independent reflections
1135 reflections with *I* > 2σ(*I*)
*R*_int_ = 0.021

#### Refinement

*R*[*F* ^2^ > 2σ(*F* ^2^)] = 0.039
*wR*(*F* ^2^) = 0.115
*S* = 1.03
1654 reflections
119 parameters
H-atom parameters constrained
Δρ_max_ = 0.15 e Å^−3^
Δρ_min_ = −0.13 e Å^−3^

### 

Data collection: *SMART* (Bruker, 2000 ▶); cell refinement: *SAINT* (Bruker, 2000 ▶); data reduction: *SAINT*; program(s) used to solve structure: *SHELXS97* (Sheldrick, 2008 ▶); program(s) used to refine structure: *SHELXL97* (Sheldrick, 2008 ▶); molecular graphics: *SHELXTL* (Sheldrick, 2008 ▶); software used to prepare material for publication: *SHELXTL*.

## Supplementary Material

Crystal structure: contains datablocks I, global. DOI: 10.1107/S1600536809003997/bi2343sup1.cif

Structure factors: contains datablocks I. DOI: 10.1107/S1600536809003997/bi2343Isup2.hkl

Additional supplementary materials:  crystallographic information; 3D view; checkCIF report

## Figures and Tables

**Fig. 1.**
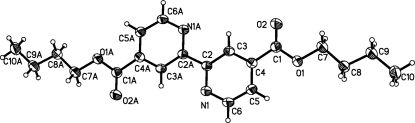
Molecular structure showing 30% probability displacement ellipsoids for non-H atoms.

## supplementary crystallographic information

### Comment

The crystal structure of 2,2'-bipyridine-4,4'-dicarboxylic acid (H_2_dcbp) has been reported by Tynan *et al.* (2003), and a polymeric structure contaning trimethyltin has been reported by Stocco *et al.* (1996). Herein, we have reacted H_2_dcbp with tri-*n*-butyltin chloride. Unexpectedly, we obtained the centrosymmetric title compound only. The C2—N1—C6 bond angle of 117.47 (15)° is similar to those for the free pyridine (Fujihara *et al.*, 2004). The dihedral angle between the pyridine ring and the carboxylate group [C1,O1,O2] is 4.37 (2)°. The bond lengths of C1—O1 and C7—O1 are 1.332 (2) and 1.458 (2) Å, respectively.

### Experimental

The reaction was carried out under a nitrogen atmosphere. 2,2'-Bipyridine-4,4'-dicarboxylic acid (1 mmol) and sodium ethoxide (2 mmol) were added to a stirred solution of benzene (30 ml) in a Schlenk flask and stirred for 0.5 h. Tri-*n*-butyltin chloride (2 mmol) was then added and the reaction mixture was stirred for 12 h at 353 K. The resulting clear solution was evaporated under vacuum. The product was crystallized from dichloromethane to yield colourless blocks (yield 83%. m.p. 435 K). Elemental analysis calculated: C, 67.10; H, 6.79; N, 7.86 %; found: C, 66.92; H, 6.95; N, 7.59 %.

### Refinement

H atoms were placed geometrically and treated as riding on their parent atoms with C—H = 0.93 Å (pyridine), 0.97 Å (methylene) or 0.96 Å (methyl), and with *U*_iso_(H) = 1.2 or 1.5*U*_eq_(C).

### Crystal data

**Table:**

C_20_H_24_N_2_O_4_	*F*(000) = 380
*M**_r_* = 356.41	*D*_x_ = 1.255 Mg m^−^^3^
Monoclinic, *P*2_1_/*c*	Mo *K*α radiation, λ = 0.71073 Å
Hall symbol: -P 2ybc	Cell parameters from 1638 reflections
*a* = 7.4183 (9) Å	θ = 2.7–26.7°
*b* = 8.2829 (10) Å	µ = 0.09 mm^−^^1^
*c* = 15.375 (2) Å	*T* = 298 K
β = 93.273 (1)°	Block, colorless
*V* = 943.2 (2) Å^3^	0.40 × 0.30 × 0.15 mm
*Z* = 2	

### Data collection

**Table:**

Bruker SMART CCD diffractometer	1654 independent reflections
Radiation source: fine-focus sealed tube	1135 reflections with *I* > 2σ(*I*)
graphite	*R*_int_ = 0.021
φ and ω scans	θ_max_ = 25.0°, θ_min_ = 2.7°
Absorption correction: multi-scan (*SADABS*; Sheldrick, 2003)	*h* = −8→8
*T*_min_ = 0.966, *T*_max_ = 0.987	*k* = −9→8
4552 measured reflections	*l* = −18→18

### Refinement

**Table:**

Refinement on *F*^2^	Primary atom site location: structure-invariant direct methods
Least-squares matrix: full	Secondary atom site location: difference Fourier map
*R*[*F*^2^ > 2σ(*F*^2^)] = 0.039	Hydrogen site location: inferred from neighbouring sites
*wR*(*F*^2^) = 0.115	H-atom parameters constrained
*S* = 1.03	*w* = 1/[σ^2^(*F*_o_^2^) + (0.0497*P*)^2^ + 0.199*P*] where *P* = (*F*_o_^2^ + 2*F*_c_^2^)/3
1654 reflections	(Δ/σ)_max_ < 0.001
119 parameters	Δρ_max_ = 0.15 e Å^−^^3^
0 restraints	Δρ_min_ = −0.13 e Å^−^^3^

### Special details

**Table:**

Geometry. All e.s.d.'s (except the e.s.d. in the dihedral angle between two l.s. planes) are estimated using the full covariance matrix. The cell e.s.d.'s are taken into account individually in the estimation of e.s.d.'s in distances, angles and torsion angles; correlations between e.s.d.'s in cell parameters are only used when they are defined by crystal symmetry. An approximate (isotropic) treatment of cell e.s.d.'s is used for estimating e.s.d.'s involving l.s. planes.
Refinement. Refinement of *F*^2^ against ALL reflections. The weighted *R*-factor *wR* and goodness of fit *S* are based on *F*^2^, conventional *R*-factors *R* are based on *F*, with *F* set to zero for negative *F*^2^. The threshold expression of *F*^2^ > σ(*F*^2^) is used only for calculating *R*-factors(gt) *etc*. and is not relevant to the choice of reflections for refinement. *R*-factors based on *F*^2^ are statistically about twice as large as those based on *F*, and *R*- factors based on ALL data will be even larger.

### Fractional atomic coordinates and isotropic or equivalent isotropic displacement parameters (Å^2^)

**Table:**

	*x*	*y*	*z*	*U*_iso_*/*U*_eq_	
N1	0.8610 (2)	0.42720 (18)	−0.09153 (9)	0.0544 (4)	
O1	0.38773 (17)	0.20544 (16)	0.09033 (7)	0.0619 (4)	
O2	0.57083 (19)	0.32666 (19)	0.19126 (8)	0.0755 (5)	
C1	0.5333 (2)	0.2904 (2)	0.11660 (11)	0.0539 (5)	
C2	0.9120 (2)	0.45867 (19)	−0.00815 (10)	0.0451 (4)	
C3	0.8062 (2)	0.4163 (2)	0.06010 (10)	0.0481 (4)	
H3	0.8437	0.4419	0.1172	0.058*	
C4	0.6451 (2)	0.3358 (2)	0.04254 (10)	0.0469 (4)	
C5	0.5929 (2)	0.3018 (2)	−0.04371 (11)	0.0540 (5)	
H5	0.4857	0.2474	−0.0581	0.065*	
C6	0.7040 (3)	0.3510 (2)	−0.10735 (11)	0.0588 (5)	
H6	0.6674	0.3297	−0.1651	0.071*	
C7	0.2671 (3)	0.1586 (3)	0.15756 (12)	0.0706 (6)	
H7A	0.2245	0.2538	0.1869	0.085*	
H7B	0.3307	0.0905	0.2005	0.085*	
C8	0.1112 (2)	0.0691 (2)	0.11506 (11)	0.0567 (5)	
H8A	0.1554	−0.0272	0.0873	0.068*	
H8B	0.0530	0.1364	0.0701	0.068*	
C9	−0.0258 (3)	0.0211 (3)	0.17920 (13)	0.0760 (6)	
H9A	−0.0695	0.1177	0.2067	0.091*	
H9B	0.0335	−0.0451	0.2244	0.091*	
C10	−0.1854 (3)	−0.0711 (3)	0.13862 (14)	0.0768 (6)	
H10A	−0.2392	−0.0103	0.0907	0.115*	
H10B	−0.2729	−0.0876	0.1814	0.115*	
H10C	−0.1455	−0.1737	0.1180	0.115*	

### Atomic displacement parameters (Å^2^)

**Table:**

	*U*^11^	*U*^22^	*U*^33^	*U*^12^	*U*^13^	*U*^23^
N1	0.0561 (9)	0.0655 (10)	0.0412 (8)	−0.0060 (8)	−0.0003 (7)	−0.0020 (7)
O1	0.0569 (8)	0.0808 (9)	0.0486 (7)	−0.0149 (7)	0.0077 (6)	0.0001 (6)
O2	0.0741 (10)	0.1073 (12)	0.0448 (7)	−0.0210 (8)	0.0004 (7)	−0.0002 (7)
C1	0.0511 (11)	0.0601 (12)	0.0500 (11)	−0.0004 (9)	−0.0006 (9)	0.0040 (9)
C2	0.0488 (9)	0.0461 (10)	0.0402 (9)	0.0038 (8)	−0.0008 (7)	0.0024 (7)
C3	0.0500 (10)	0.0538 (11)	0.0396 (9)	0.0026 (8)	−0.0048 (8)	0.0012 (8)
C4	0.0467 (10)	0.0489 (10)	0.0449 (9)	0.0053 (8)	0.0012 (7)	0.0029 (8)
C5	0.0516 (11)	0.0598 (11)	0.0499 (10)	−0.0042 (9)	−0.0028 (8)	−0.0042 (9)
C6	0.0627 (12)	0.0733 (13)	0.0396 (9)	−0.0066 (10)	−0.0036 (9)	−0.0054 (9)
C7	0.0682 (13)	0.0945 (16)	0.0497 (11)	−0.0170 (11)	0.0091 (10)	0.0039 (11)
C8	0.0573 (11)	0.0626 (12)	0.0506 (10)	−0.0001 (9)	0.0064 (9)	0.0042 (9)
C9	0.0731 (14)	0.1050 (17)	0.0507 (11)	−0.0187 (13)	0.0097 (10)	0.0063 (11)
C10	0.0684 (14)	0.0923 (16)	0.0705 (13)	−0.0141 (12)	0.0096 (11)	0.0132 (12)

### Geometric parameters (Å, °)

**Table:**

N1—C6	1.335 (2)	C6—H6	0.930
N1—C2	1.341 (2)	C7—C8	1.493 (3)
O1—C1	1.332 (2)	C7—H7A	0.970
O1—C7	1.458 (2)	C7—H7B	0.970
O2—C1	1.204 (2)	C8—C9	1.509 (3)
C1—C4	1.495 (2)	C8—H8A	0.970
C2—C3	1.391 (2)	C8—H8B	0.970
C2—C2^i^	1.483 (3)	C9—C10	1.513 (3)
C3—C4	1.381 (2)	C9—H9A	0.970
C3—H3	0.930	C9—H9B	0.970
C4—C5	1.389 (2)	C10—H10A	0.960
C5—C6	1.377 (2)	C10—H10B	0.960
C5—H5	0.930	C10—H10C	0.960
			
C6—N1—C2	117.47 (15)	C8—C7—H7A	110.0
C1—O1—C7	116.43 (14)	O1—C7—H7B	110.0
O2—C1—O1	124.07 (17)	C8—C7—H7B	110.0
O2—C1—C4	123.74 (17)	H7A—C7—H7B	108.4
O1—C1—C4	112.18 (15)	C7—C8—C9	112.24 (16)
N1—C2—C3	122.14 (15)	C7—C8—H8A	109.2
N1—C2—C2^i^	116.64 (18)	C9—C8—H8A	109.2
C3—C2—C2^i^	121.23 (17)	C7—C8—H8B	109.2
C4—C3—C2	119.56 (15)	C9—C8—H8B	109.2
C4—C3—H3	120.2	H8A—C8—H8B	107.9
C2—C3—H3	120.2	C8—C9—C10	113.81 (17)
C3—C4—C5	118.40 (16)	C8—C9—H9A	108.8
C3—C4—C1	118.96 (15)	C10—C9—H9A	108.8
C5—C4—C1	122.64 (16)	C8—C9—H9B	108.8
C6—C5—C4	118.21 (17)	C10—C9—H9B	108.8
C6—C5—H5	120.9	H9A—C9—H9B	107.7
C4—C5—H5	120.9	C9—C10—H10A	109.5
N1—C6—C5	124.20 (16)	C9—C10—H10B	109.5
N1—C6—H6	117.9	H10A—C10—H10B	109.5
C5—C6—H6	117.9	C9—C10—H10C	109.5
O1—C7—C8	108.26 (15)	H10A—C10—H10C	109.5
O1—C7—H7A	110.0	H10B—C10—H10C	109.5
			
C7—O1—C1—O2	1.5 (3)	O2—C1—C4—C5	−175.64 (18)
C7—O1—C1—C4	−178.51 (15)	O1—C1—C4—C5	4.4 (2)
C6—N1—C2—C3	−0.8 (3)	C3—C4—C5—C6	−0.4 (3)
C6—N1—C2—C2^i^	179.14 (18)	C1—C4—C5—C6	178.98 (16)
N1—C2—C3—C4	1.5 (3)	C2—N1—C6—C5	−0.6 (3)
C2^i^—C2—C3—C4	−178.40 (18)	C4—C5—C6—N1	1.2 (3)
C2—C3—C4—C5	−0.9 (2)	C1—O1—C7—C8	178.49 (16)
C2—C3—C4—C1	179.76 (15)	O1—C7—C8—C9	−177.70 (17)
O2—C1—C4—C3	3.7 (3)	C7—C8—C9—C10	−179.62 (19)
O1—C1—C4—C3	−176.26 (15)		

Symmetry codes: (i) −*x*+2, −*y*+1, −*z*.
